# Allogeneic hematopoietic stem cell transplantation as the first-line treatment option in a patient with severe aplastic anemia without a matched related donor: A case report

**DOI:** 10.3892/ol.2014.2341

**Published:** 2014-07-10

**Authors:** SHUN-NENG HSU, JIA-HONG CHEN, WOEI-YAU KAO

**Affiliations:** 1 Division of Hematology-Oncology, Department of Medicine, Tri-Service General Hospital, National Defense Medical Center, Taipei 114, Taiwan, R.O.C; 2 Division of Hematology-Oncology, Department of Medicine, Buddhist Tzu Chi General Hospital, Taipei 231, Taiwan, R.O.C

**Keywords:** unrelated donor, peripheral blood transplants, allogeneic hematopoietic stem cell transplantation, aplastic anemia, bone marrow transplants

## Abstract

The outcomes of matched unrelated donor (MUD) hematopoietic stem cell transplantation (HSCT) and immunosuppressive therapy (IST) in patients with severe aplastic anemia (SAA) remain controversial. The clinical outcome in patients that undergo transplantation following failed IST is typically poorer when compared with patients that initially underwent transplantation. Clinical treatment algorithms have been proposed to determine the management of such patients, and account for individual conditions, personal preferences and prognostic risk factors. The present study reports the promising outcome of a 22-year-old patient exhibiting SAA. The patient underwent peripheral blood stem cell transplantation (PBSCT) from an MUD using a fludarabine-based conditioning regimen and low-dose total body irradiation as an alternative method to first-line IST. The patient achieved rapid bone marrow reconstitution and has been in complete remission for 32 months. The aim of the fludarabine-based conditioning regimen with PBSCT was to improve the patient’s therapeutic outcome and provide a convenient treatment strategy. Furthermore, this regimen extends the application of HSCT to patients who are older or those that are without a matched related donor.

## Introduction

Severe aplastic anemia (SAA) is a rare and potentially fatal disease that is characterized by an immune-mediated functional impairment of hematopoietic stem cells and predominantly affects young adults (age range, 20–39 years) (1). The treatment of SAA includes immunosuppressive therapy (IST) and hematopoietic stem cell transplantation (HSCT) (2,3). IST with antithymocyte globulin (ATG) and cyclosporine (CsA) is the first-line therapy in children and young adults who are unsuitable candidates for HSCT or without a suitable donor. HSCT, with peripheral blood stem cell transplantation (PBSCT) or bone marrow transplantation (BMT), is the treatment of choice for young patients who have a matched related donor (MRD). A number of previous studies have demonstrated successful HSCTs using a matched unrelated donor (MUD), with outcomes similar to those of patients who underwent MRD HSCT. This treatment option is currently reserved for patients who do not respond to IST, exhibit relapse or develop secondary clonal disorders following IST (4). A recent review demonstrated that an MUD HSCT may initially be considered for the treatment of children without an MRD (5). However, the appropriate definition of the lower age limit for HSCT varies considerably across studies. Investigations into extending the application of HSCT to patients who are older or without an MRD are currently in progress (6). Patient provided written informed consent.

## Case report

**Figure 1:**
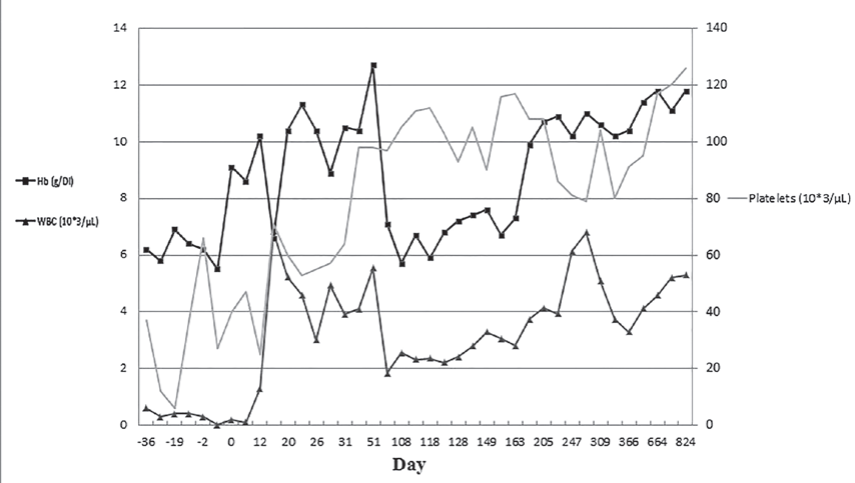
Cell count changes in the peripheral blood following engraftment. Hb, hemoglobin; WBC, white blood cell.

On 20th March, 2011, a 22-year-old male presented to the Tri-Service General Hospital (Taipei, Taiwan) with a three-month history of SAA. Laboratory examinations prior to non-myeloablative HSCT showed the following abnormalities: Leukocyte count, 0.3×10^3^/μl (reference range, 4.5–11.0×10^3^/μl); hemoglobin level, 5.8 g/dl (reference range, 12.0–16.0 g/dl); and platelet count, 12×10^3^/μl (reference range, 150–400×10^3^/μl). Subsequent bone marrow examination revealed hypocellularity (<20% of cellular bone marrow) with 10% of that, which is typically observed in healthy young adults. An MUD was successfully identified with a human leukocyte antigen (HLA) major antigen match (8/8 loci) for the patient, from the Tzu Chi Stem Cells Center (Hualien, Taiwan). The conditioning regimen comprised fludarabine (30 mg/m^2^), administered intravenously daily for four days (six to three days prior to the transplantation); cyclophosphamide (300 mg/m^2^), administered intravenously daily for four days (six to three days prior to the transplantation); and ATG (3.75 mg/kg), administered intravenously for three days (five to three days prior to the transplantation) as a 12-h infusion. The patient underwent low-dose total body irradiation (TBI; 200 cGy) on one day prior to receiving an allograft infusion. The number of cluster of differentiation 34^+^ cells infused per kilogram of recipient weight was 6.6×10^6^ (total, 410.99×10^6^ nucleated cells), with a major ABO blood group mismatch between the recipient (type O+) and donor (type A+). The patient and the donor tested negative for hepatitis B and C viruses, cytomegalovirus and human immunodeficiency virus, as determined by serological tests. Graft-versus-host disease (GVHD) prophylaxis consisted of methotrexate and CsA. Methotrexate was administered intravenously at 10 mg/m^2^ one day following the transplantation and 8 mg/m^2^ three and six days following the transplantation. CsA was administered intravenously at 1 mg/kg as a 12-h infusion on days one, three and six following the transplantation. The dose of CsA was adjusted depending on the presence of a skin rash, as well as liver function. The patient also received filgrastim at a dose of 5 μg/kg/day intravenously, from day one following the transplantation until the white blood cell count exceeded 4×10^3^/μl or the neutrophil count exceeded 0.5×10^3^/μl on day 14 following the transplantation. The absolute neutrophil count (ANC) of 1.079×10^3^/μl (reference range, 1.5–8.0×10^3^/μl) was achieved with a platelet count of >25×10^3^/μl from day 12 following the transplantation for three consecutive days. The donor origin of engraftment was confirmed by polymerase chain reaction analysis of short tandem repeats on day 20 following the transplantation and >99% of donor hematopoiesis was recorded in the patient with blood type A. Following successful engraftment 108 days following the transplantation, the hemoglobin level decreased to 5.7 mg/dl, therefore, the CsA dose was adjusted and the ANC and hemoglobin levels gradually increased to within the normal limits without a blood transfusion. The cell count changes of the peripheral blood for the patient are demonstrated in Fig. 1. The preparative regimen was well tolerated and regimen-associated toxicity, including anorexia and enteritis, was mild. No complications involving any infectious diseases occurred in the patient. However, symptoms of acute GVHD were observed on day 10 following the transplantation with a grade I generalized skin rash and abnormal liver function tests, indicating an elevation of aminotransferases. A single dose of methylprednisolone (40 mg) was administered, and subsequently discontinued following the return of normal liver function. However, the sustained engraftment immunosuppressive agent with low dose cyclosporine (50 mg per day) was continued. No recurrence of acute GVHD occurred and to date, chronic GVHD has not been observed. In addition, a good performance status has been observed for >32 months.

## Discussion

The treatment of SAA predominantly comprises IST and allogeneic HSCT (2,3,7–9), in which allogeneic HSCT is considered the first-line treatment for young patients with an MRD available, and if the patient is <40 years of age (8,10–13). However, >70% of patients do not have an MRD (2,14). However, the appropriate definition of the lower age limit for HSCT varies considerably across studies. Clinical treatment algorithms have been proposed to determine the management of such patients, and account for individual conditions, personal preferences and prognostic risk factors (15). Based on these difficulties, the aim of the present study was to extend the application of HSCT to patients who are older or without an MRD.

In previous studies, MUD HSCT has been regarded by the majority of clinicians as the follow-up option for patients who failed to respond to a course of IST (1,5,14,16). Furthermore, various studies have demonstrated that HSCT-associated toxicity may be mitigated by careful selection of patients (e.g. recipient age, time interval between diagnosis and transplantation, and performance status), donors (determined by detailed HLA matching), the conditioning regimen (fludarabine-based regimens) and improved supportive care (1,5,17). Avoidance of IST and multiple transfusions prior to transplantation has been reported as a prerequisite for achieving improved survival rates (15). Furthermore, BMT is considered to have a significant advantage over IST with regard to survival rate and the risk of relapse (4,18). The consensus from previous studies is that bone marrow must be used as it is the preferred stem cell source, particularly when a patient is undergoing an MRD HSCT (19,20). When the donor is an MUD, the use of peripheral blood can be proposed as the graft source (21). In Taiwan, HSCT has become the conventional therapy for treating patients with hematological diseases following the initiation of the Buddhist Tzu Chi Marrow Donor Registry in October 1993, using graft types, including peripheral blood (60.4%) and bone marrow stem cells (32.0%) (22,23). In the present case, varying combinations of the conditioning regimen were used, which comprised of fludarabine, low-dose cyclophosphamide and ATG, with low-dose TBI (15). This regimen, in combination with MUD PBSCT, was administered to a carefully selected SAA patient, with the aim of improving the patient outcome using a convenient treatment strategy.

In conclusion, in carefully selected, young patients, it may be appropriate to perform HSCT as the first-line therapy, even when a matched donor is identified, due to the likelihood of greater long-term efficacy and rapid engraftment. The patient described in the present study has been in complete remission with a good performance status for >32 months and, therefore, provides a demonstration of the feasibility of this approach. The current study indicates that a PBSCT from an MUD may deliver a promising and curative outcome in young patients without an MRD.
